# Aneuploidy screening using circulating fetal cells in maternal blood by dual‐probe FISH protocol: a prospective feasibility study on a series of 172 pregnant women

**DOI:** 10.1002/mgg3.249

**Published:** 2016-10-26

**Authors:** Giuseppe Calabrese, Donatella Fantasia, Melissa Alfonsi, Elisena Morizio, Claudio Celentano, Paolo Guanciali Franchi, Giulia Sabbatinelli, Chiara Palka, Peter Benn, Gianmaria Sitar

**Affiliations:** ^1^ Genetica MedicaUniversità Chieti‐PescaraChieti ScaloItaly; ^2^ Clinica Ostetrica e GinecologicaASL ChietiChietiItaly; ^3^ University of Connecticut Health CenterFarmingtonConnecticut; ^4^ Policlinico S. MatteoPaviaItaly

**Keywords:** Aneuploidy, circulating fetal cells, FISH, genetic counseling, NIPT

## Abstract

**Background:**

A long sought goal in medical genetics has been the replacement of invasive procedures for the detection of chromosomal aneuploidies by isolating and analyzing fetal cells or free fetal DNA from maternal blood, avoiding risk to the fetus. However, a rapid, simple, consistent, and low‐cost procedure suitable for routine clinical practice has not yet been achieved. The purpose of this study was to assess the feasibility of predicting fetal aneuploidy by applying our recently established dual‐probe FISH protocol to fetal cells isolated and enriched from maternal blood.

**Methods:**

A total of 172 pregnant women underwent prospective testing for fetal aneuploidy by FISH analysis of fetal cells isolated from maternal blood. Results were compared with the karyotype determined through invasive procedures or at birth.

**Results:**

Seven of the samples exhibited fetal aneuploidy, which was confirmed by invasive prenatal diagnosis procedures. After enrichment for fetal cells, the frequency of trisomic cells was at least double in samples from aneuploid pregnancies (range 0.38–0.90%) compared to samples from normal pregnancies (≤0.18%). One false negative result was also obtained.

**Conclusions:**

Noninvasive prenatal aneuploidy screening using fetal cells isolated from maternal blood is feasible and could substantially reduce the need for invasive procedures.

## Introduction

Prenatal genetic diagnosis currently relies on the examination of fetal cells obtained through invasive procedures, such as amniocentesis and chorionic villous sampling. These procedures are associated with a risk to the pregnancy. A long sought goal in medical genetics has been the replacement of invasive procedures by testing isolated fetal cells or free fetal DNA from maternal blood. Although substantial progress has been made in the development of tests based on the presence of cell‐free fetal DNA in maternal plasma, a rapid, simple, consistent, and low‐cost procedure suitable for routine clinical practice for all women has not yet been achieved (Takabayashi et al. 1995; Bianchi et al. 1997; Bischoff et al. 1998 Jul; Fan et al. 2008; Benn et al. 2013; Cuckle et al. 2015; Gil et al. 2015). Krabchi et al. (2001) offered conclusive data that fetal cells are always present in maternal blood, their numbers being 2–6 fetal cells/mL maternal blood, which is equivalent to 1 fetal cell in 1–3 × million nucleated maternal cells or 1 fetal cell in 1 × 1–2 billion red blood cells. The very low number of fetal cells in maternal blood represents the most formidable obstacle for reliable genetic testing (Evans and Kilpatrick 2010; Fiddler 2014). In this context, the finding of fetal cells from prior pregnancies in maternal circulation (Bianchi et al. 1996) appears as an exceptional event (accounting for 1–3 cells in 20 mL maternal blood) with very limited effect, if any, on the fetal cell search from the current pregnancy (Coata et al. 2001, 2009; Guetta et al. 2003).

We previously described a method for enriching fetal cells from maternal blood, including epsilon‐Hb‐positive nucleated red blood cells (NRBCs), i‐positive, and CD34‐positive cells (Sitar et al. 2005). A fluorescence in situ hybridization (FISH) analysis of these fetal cell‐enriched samples was carried out using two independent probes for chromosomes 18 and 21 (Calabrese et al. 2012). A correct diagnosis was achieved for all pregnancies; the mean percentage of trisomic cells was 0.5% (range 0.36–0.76%) in the aneuploidy group compared with ≤0.20% in the control group (normal pregnancies or nonpregnant women). Using these procedures, we herein report results for a prospective study of 172 pregnant women.

## Materials and Methods

### Samples

The written informed consents were obtained according to the local guidelines for genetic studies, and were in accordance with the ethical guidelines of the Declaration of Helsinki. After genetic counseling and informed consent, blood samples (24 mL) were collected from 172 consecutive women with singleton pregnancies attending an antenatal clinic where they underwent aneuploidy screening (serum biochemical assays), and at least 7 days prior to any invasive prenatal diagnostic (IPD) procedures. The average maternal age was 36.1 years (range 24–45 years), and average gestational age at sampling was 12.5 weeks (range 10–18). Twenty‐four women underwent IPD procedures because of advanced maternal age (≥35 years, AMA), and two because of a familial balanced translocation (one translocation t(10;18)pat and one Robertsonian translocation rob(13;14)pat). Of the remaining women, 79 were determined to be at low risk (LR; <1/900), 38 at intermediate risk (IR; 1:31 to 1:899), and 29 at high risk (HR; ≥1:30) for fetal aneuploidy according to a contingent screening protocol (Guanciali‐Franchi et al. 2011, 2012). Thirty‐two women with positive biochemical screening results underwent an IPD procedure. Ultrasonographic evidence of fetal anomalies was present in 20 out of 165 (12.3%) normal pregnancies and in 6 out of 7 (86%) pregnancies with aneuploid fetuses. The average maternal body weight was 59 kg (range 45–105 kg).

As of 28 February 2016, 164 women had delivered a normal baby and eight had terminated their pregnancy due to the IPD finding of an aneuploid fetus. The physical examination and cytogenetics on newborns determined that aneuploidy was not present in the live births.

All fetal cell analyses were completed before the chromosome analyses were available. The primary end point was the detection rate and false positive rate for the detection of fetal trisomies 21 and 18 achieved through fetal cell analysis with pregnancy outcomes or prenatal karyotypes as the reference standard.

### Fetal cell isolation

Maternal blood samples were immediately transferred into nonphysiological conditions and left overnight before separation by density gradient centrifugation as previously described (Sitar et al. 2005). Briefly, maternal blood samples were mixed with an equal volume of 1× medium‐199 with Earle salts (Sigma‐Aldrich, St Louis, MO) and immediately, 15% of ACD‐A was added to blood samples. The osmolarity of these solutions was adjusted to 320 mOsm/L using NaCl (20 mEq/10 mL).

Diluted blood samples were overlaid onto a Biocoll solution (Biochrom AG, Berlin, Germany) having a density of 1.072 g/L into a cell separation device. Centrifugation was run for 20 min at 400 × g. Cells floating at the interface were retrieved out of the separation device previously described (Sitar et al. 2005). Slides for FISH investigation were obtained by cytocentrifugation. The procedure took about 2 h with a capacity of four samples per run.

### Dual‐labeling FISH‐based detection

Fluorescence in situ hybridization investigation was performed according to the procedure previously described dual‐probe FISH analysis; ref. Calabrese et al. 2012). Using the protocol described by Yan et al. (2000) which removes the cytoplasm and swells nuclei thus facilitating FISH analysis, cells from enriched samples were incubated in prewarmed (37°C) hypotonic KCl (0.075 mol/L) for 5 min and fixed three times with cold Carnoy's fixative (methanol:glacial acetic acid, 3:1) before cytocentrifugation on glass slides. To investigate chromosomes 18 and 21, FISH experiments were carried out using a genomic single copy probe specific for the q arm (Kreatech‐Resnova, Rome, Italy) combined with a subtelomeric probe (Cytocell‐Euroclone, Milan, Italy), for each chromosome, each probe labeled with FITC and with Texas Red, respectively. Slides were pretreated with pepsin (0.01 N HCl; Sigma‐Aldrich) for 5 min and then dehydrated in an ethanol series. Slides were observed under a fluorescence microscope by direct visualization. At least 2000 mononuclear cells per sample were scored (2000–9600 scored cells by direct visualization using an appropriate triple pass‐band filter (Zeiss, Jena, Germany). Cells showing a two green/two red signal FISH pattern were classified as normal, whereas cells with a three green/three red signal pattern were classified as trisomic. All other patterns of hybridization were excluded from the analysis, although they were recorded for hybridization quality control (Calabrese et al. 2012). Multinuclear cells, representing exceptional findings, were excluded in the FISH scoring process to avoid FISH signals misinterpretation.

### Statistics

The Mann–Whitney *U*‐test was used to evaluate the difference in the percentage of trisomic cells between aneuploid pregnancies versus normal pregnancies.

## Results

Of the 172 cases tested, 164 were normal pregnancies and eight were abnormal pregnancies (five cases of trisomy 21, two of trisomy 18, and one triploid; Table 1). After fetal cell isolation, 160,000–220,000 cells were recovered from each 24 ml maternal blood sample. No significant correlations were found between the cell yield and maternal age or risk based on screening, and no difference was found between euploid and chromosomally abnormal cases.

**Table 1:** Fluorescence in situ hybridization on enriched fetal samples (fcNIPT) and IPD karyotyping data in pregnant women with different aneuploidy risk levels

Risk levels	Euploid	+21		+18		Triploidy		Total
	Pregnancies	fcNIPT	IPD	fcNIPT	IPD	fcNIPT	IPD	
Parental translocation	2	0	0	0	0	0	0	2
AMA	23	1	1	0	0	0	0	24
LR	78	0	0	1	1	0	0	79
IR	36	1	1	0	0	1	1	38
HR	26	2^a^	3	1	1	0	0	29
Total	165	4	5	2	2	1	1	172

AMA, advanced maternal age (≥35aa); LR, low risk (≤1:900); IR, intermediate risk (1:31/1:899); HR, high risk (≥1:30).

a False negative fcNIPT result (trisomy 21 from rob(14;21)mat).

For the 165 cases with normal karyotypes in FISH (Table 1), 390,500 nuclei were scored for chromosome 18 FISH signals, with 0.52/1000 (range 0–1.8/1000) cells showing evidence of trisomy 18. For cases in which one or more cell was considered to be trisomic, the average number of cells with apparent trisomy 18 was 0.72/1000 (range 0.3–1.8/1000 cells). Similarly, for the normal cases based on FISH, chromosome 21 signals were evaluated in 396,343 nuclei with 0.89/1000 cells apparently showing trisomy 21 (range 0–1.7/1000 cells). Among these euploid cases with at least one cell scored with apparent trisomy 21, the average number of trisomy 21 cells was 1.02/1000 (range 0.3–1.7/1000 cells). In all normal FISH samples, scoring for trisomies 18 and 21 was ≤1.8/1000 and ≤1.7/1000 analyzed cells, respectively. The proportion of cells with three signals was significantly higher among the seven cases with aneuploidy than the cases with apparently normal karyotypes (*P* < 0.01).

**Figure 1:**
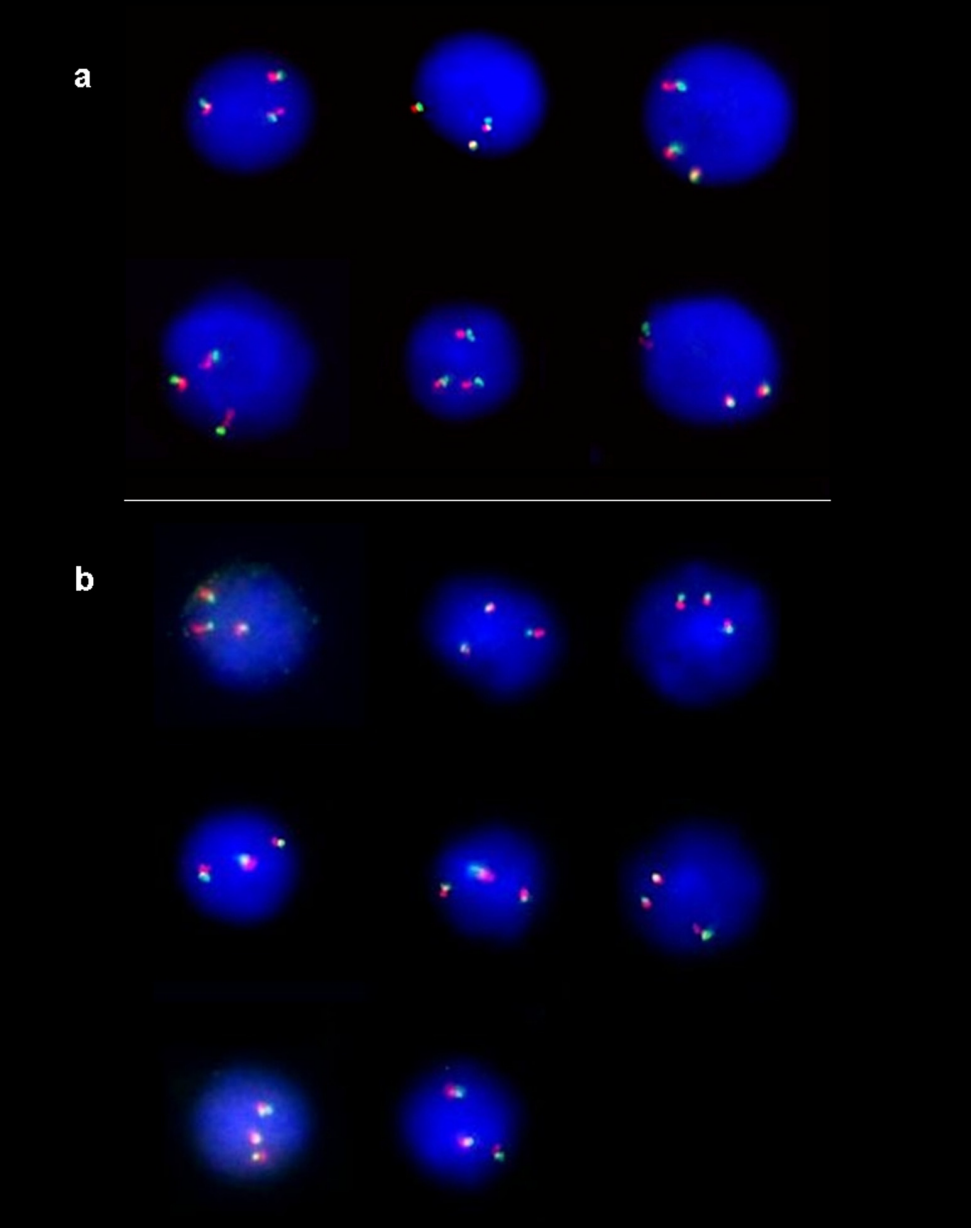
(A) Dual‐probe FISH analysis using two probes for different loci on chromosome 21 showing three green and three red signals in cells from a pregnant women carrying a trisomy 21 fetus (patient B42). Yellow signals results from overlapping of green and red signals. (B) Dual‐probe FISH analysis using two probes for different loci on chromosome 18 showing three signals in green and three signals in red in cells from a pregnant women with a trisomy 18 fetus (patient C46). Yellow signals results from overlapping of green and red signals.

By FISH analysis, seven samples showed ≥3.8/1000 cells consistent with aneuploidy (Fig. 1; Table 2). IPD procedures confirmed aneuploidy in these cases (four cases of trisomy 21, two of trisomy 18, and one triploidy 69, XXY). In these seven samples with confirmed abnormal karyotypes, four cases of trisomy 21 had 6, 6, 8, and 9 positive cells in 1000 scored, respectively (Table 2); the two cases of trisomy 18 had 3.8 and 8 positive cells in 1000 cells, respectively; and in the one triploidy case trisomy 21 cells were 8 positive cells in 1000, and trisomy 18 cells were 9 positive cells in 1000.

**Table 2:** Samples with aneuploidy detected cells at borderline or above real trisomy cut‐off (trisomy cells in aneuploid fetus ≥3.6/1000; trisomy cells in normal fetus ≤2/1000; ref. Calabrese et al 2012)

Pat. no.	Age	GA	Risk	FISH (Cells+/1000)	Scored cells	IPD	Maternal weight (kg)	
A14	41	14	AMA	+18 0/2000 +21 **6/1000**	3000	AF 47,XX,+21	56.5	Aneuploidy group
B42	29	13	Cystic hygrome NT3,5 HR +21	+18 0/2000 +21 **9/1000**	3000	AF 47,XX,+21	45	
D23	32	12	HR +21	+18 0/2000 +21 **8/1000**	3000	CVS 47,XX,+21	57	
D55	38	13 + 3	HR +21	+18 0/2000 +21 **6/1000**	3000	CVS 47,XY,+21	62	
B43	27	12 + 5	NT 2,81 LR	+18 **8/1000** +21 1/3000 *(0.3/1000)*	4000	CVS 47,XY,+18	58	
C46	19	11 + 4	US fetal anomalies HR +18	+18 8/2600 ***(3.8/1000)*** +21 0/2000	4600	CVS 47,XX,+18	56	
B8	24	18	HR +18	+18 **8/1000** +21 **9/1000**	2000	AF 69,XXY	59	
C72	37	13 + 5	HR +21	+18 0/4000 +21 9/5600 *(1.7/1000)*	9600	AF 46,XY	58	Diploidy group
C87	39	12 + 4	HR +21	+18 9/5000 *(1.8/1000)* +21 1/3400 *(0.3/1000)*	8400	AF 46,XX	53.5	
C89	31	13 + 0	NT 2.8 HR +21 HR +18	+18 1/4000 *(0.25/1000)* +21 1/4000 *(0.25/1000)*	8000	AF 46,XX,rob(14;21)	68.5	False negative

In bold, trisomy cell frequency above cut‐off; in brackets, trisomy cell frequency in 1000 scored cells.

In a single case, FISH provided a normal result for both chromosomes 18 and 21 (Table 2), but the amniotic fluid karyotyping, performed after positive biochemical serum screening, disclosed trisomy 21 due to an unbalanced Robertsonian translocation rob(14;21). Cytogenetic analysis carried out in the parents showed a normal karyotype in the father, and a balanced rob(14;21) translocation in the mother.

Overall, the FISH analysis had a detection rate of 7/8 (87.5%, 95% CI 42–99%).

## Discussion

We report the results of a prospective investigation on 172 pregnant women using fetal cells isolated from maternal blood. The aim was to screen for trisomy 18 and trisomy 21, and triploidy which represent 61% of prenatal cytogenetic abnormalities identifiable by conventional karyotyping of invasive test samples and are the main target for current noninvasive prenatal testing (NIPT) (Snijders et al. 1998; Grati et al. 2010).

Seven of the samples had evidence of fetal aneuploidy that was confirmed by IPD procedures. In positive pregnancies, trisomy cells occurred at least twice as frequently than in samples from normal pregnancies. However, in one case, the FISH analysis was unable to detect trisomic cells. False‐negatives may be obtained when the fetal cell population is very low. The main origin of the circulating fetal cells is thought to be placental tissues (Bianchi and Robert 2007; Klonisch and Drouin 2010); therefore, additional studies on fetal and placental tissue could help in determining the causes of discordant FISH results in order to prevent or avoid this harmful event.

In our experience a stringent adherence to the hybridization protocol from Yan et al. (2000), including a 5 min KCl hypotonic treatment, combined with dual‐probe FISH protocol (Kilpatrick et al. 2004; Mergenthaler et al. 2005; Fiddler 2014) was found to be optimal providing intact nuclei and unambiguous FISH signals. In the present series, the finding of very few aneuploid cells (≤0.18%) in normal pregnancies is likely due to experimental FISH artifacts, although we cannot exclude other rare events, including the presence of trisomic cells due to residual circulating fetal cells from previous aneuploid (sometimes vanishing) pregnancies, very low trisomic mosaicism, or duplication of target FISH genomic regions in the investigated subject (Lambert et al. 2005; Yan et al. 2005; Krabchi et al. 2006; Snyder et al. 2015).

This proof‐of‐principle study shows that the FISH‐based approach on fetal cell‐enriched maternal blood samples could be a feasible screening test for the selection of those pregnant women who would most benefit from IPD procedures. The test could be an adjunct to biochemical and ultrasound screening or applied to women of advanced maternal age. Currently, a large number of cells need to be scored per sample (≥2000 cells), but automated FISH microscopy analysis should speed up result delivery and laboratory productivity (Kilpatrick et al. 2004; Calabrese et al. 2012; Emad et al. 2014).

Further investigations on a larger series of samples are necessary to validate this approach. The development of a protocol for the routine isolation of fetal cells could have applications far beyond aneuploidy detection.

## Conflict of Interest

P. Benn is a consultant at Natera, Inc.
